# Treatment of Branch Retinal Vein Occlusion induced Macular Edema with Bevacizumab

**DOI:** 10.1186/1471-2415-8-18

**Published:** 2008-09-29

**Authors:** Mathias Abegg, Christoph Tappeiner, Ute Wolf-Schnurrbusch, Daniel Barthelmes, Sebastian Wolf, Johannes Fleischhauer

**Affiliations:** 1 Department of Ophthalmology, Inselspital, University of Bern, Switzerland

## Abstract

**Background:**

Branch retinal vein occlusion is a frequent cause of visual loss with currently insufficient treatment options. We evaluate the effect of Bevacizumab (Avastin^®^) treatment in patients with macular edema induced by branch retinal vein occlusion.

**Methods:**

Retrospective analysis of 32 eyes in 32 patients with fluorescein angiography proven branch retinal vein occlusion, macular edema and Bevacizumab treatment. Outcome measures were best corrected visual acuity in logMAR and central retinal thickness in OCT.

**Results:**

Visual acuity was significantly better 4 to 6 weeks after Bevacizumab treatment compared to visual acuity prior to treatment (before 0.7 ± 0.3 and after 0.5 ± 0.3; mean ± standard deviation; p < 0.01, paired t-test). Gain in visual acuity was accompanied by a significant decrease in retinal thickness (454 ± 117 to 305 ± 129 μm, p < 0.01, paired t-test). Follow up (170, 27 – 418 days; median, range) shows that improvement for both visual acuity and retinal thickness last for several months after Bevacizumab use.

**Conclusion:**

We present evidence that intravitreal Bevacizumab is an effective and lasting treatment for macular edema after branch retinal vein occlusion.

## Background

Branch retinal vein occlusion (BRVO) is a frequent retinal vascular disease with an incidence of 2.14/1000/year in the population over 40 years of age[1]. It may cause immediate vision loss due to reduced blood perfusion and subsequent retinal hypoxia. It is also often is complicated, with a temporal delay, by macular edema. The edema may cause an additional reduction in visual acuity that often exceeds the primary ischemic damage, and thus represents an important treatment target. In the past macular edema was treated with focal photocoagulation and more recently with intravitreal triamcinolone[2,3]. Both treatments show a significant but limited success. It has previously been shown that intravitreal levels of the vascular endothelial derived growth factor protein (VEGF) are significantly increased after BRVO[4] and it is currently thought that ischemia-induced upregulation of VEGF causes a loosening of tight junctions which in return results in vascular leakage and edema. We therefore sought to investigate the use of anti-VEGF therapy for treatment of BRVO-induced macular edema.

## Methods

Retrospective analysis was performed in 32 consecutive eyes from 32 patients examined in our outpatient department. 15 patients were female, 17 male. All patients had fluorescein-angiography proven BRVO and at least one injection of Bevacizumab (Avastin^®^). Patients were included independently of a prior or concurrent treatment with focal laser, intravitreal triamcinolone or hemodilution.

Examination of patients consisted of: (1) Determination of best corrected visual acuity (BCVA) using Snellen charts, (2) slit lamp examination, (3) measurement of central retinal thickness (CRT) using the Zeiss^® ^Stratus OCT (Carl Zeiss Meditec AG, Oberkochen, Germany) and (4) fluorescein angiography. BCVA was transformed into logMAR values to facilitate statistical analysis. OCT recordings were performed using the fast scan routine provided by the software. CRT (in μm) was measured in the central circle provided by the 3 dimensional data analysis tool.

Informed consent for off-label use of Bevacizumab was obtained from all patients prior to injection. Bevacizumab (1.25 mg) was injected intravitreally via pars plana under sterile conditions in the operation theatre. Patients used topical antibiotics (Tobramycin) 4 times per day for 1 week after the injection.

Primary outcome measures were changes in BCVA (in logMAR) and CRT compared to measures made at indication of treatment (referred to as 'before'). All values in this article are expressed as median and range or as mean ± standard deviation respectively, where indicated. For statistical analysis student's paired t-test was used. Spearman's rank correlation coefficient was applied for correlation analysis. A p-value of < 0.01 was considered as statistically significant.

Our study complied with the provisions of the Declaration of Helsinki and was approved by the local ethics committee.

## Results

### Baseline Characteristics

Median age of patients was 65 years, ranging from 48 to 87 years. BCVA at the time of diagnosis was 0.46 ± 0.3 logMAR. CRT was 437 ± 164 μm. Time between diagnosis and the first Bevacizumab injection ranged from 5 days to 18 years, median was 113 days. Median time interval between indication of treatment and the actual injection was 9 days, ranging from injection on the same day to 44 days later. 47% of all patients had focal laser photocoagulation, 13% intravitreal triamcinolone treatment and 6% had prior hemodilution. Additional treatments are listed in Table 1. No laser, triamcinolone or hemodilution was applied between the last consultation before and the first consultation after Bevacizumab treatment.

**Table 1:** Additional treatments

Patient	Time-to-Treat [d]	Visual Gain	Follow-up [d]	Total Injections	Focal Laser [m]	Hemodilution	Intravitreal Triamcinolone [m]	Other
1	2232	0.0	119	1	-	-	-	-
2	59	0.8	57	1	-	-	-	-
3	19	0.2	341	4	-	y	-	-
4	77	0.2	172	2	-	-	-	-
5	6715	0.3	94	1	-1	-	-	-
6	17	1.1	221	3	+4,+13	-	-	-
7	37	0.7	250	3	-	-	-	-
8	211	0.2	109	1	-	-	-	-
9	6	0.0	91	1	-	-	-	-
10	1041	0.1	193	2	-	-	-	-
11	26	-0.1	33	1	-	-	-	-
12	505	0.0	90	1	-14,-12,-9,-8,-3,-2	-	-14, +2	-
13	9	0.0	278	2	+1,+2,+8,+12	-	+9	-
14	456	0.6	236	2	+1,+2,+3,+7	-	-15	-
15	973	0.1	57	1	-27,-24	-	-	-
16	598	0.2	27	1	-8,-3	-	-3	-
17	134	0.3	418	1	-3,-2,+13	-	-	-
18	10	0.6	179	2	-	-	-	-
19	216	0.1	339	2	+1	-	-	-
20	5	-0.2	171	2	+1,+4,+6	-	-	-
21	1794	0.3	284	4	+1	-	-	-
22	5	0.3	38	1	-	-	-	-
23	64	0.2	48	1	-	y	-	-
24	254	0.1	234	4	+2,+7	-	-	-
25	626	0.1	31	1	-	-	-	-
26	5	-0.2	168	2	-	-	-	-
27	47	0.6	229	3	+6,+7,+11	-	-	-
28	92	-0.3	28	1	-3	-	-	-
29	854	0.0	81	1	-	-	-	vitrectomy at +2 m for tractive retinal detachment
30	312	0.1	360	2	-2,+7	-	-	vitrectomy at +7 m for vitreous hemorrhage
31	8	0.1	96	1	-	-	-	-
32	371	0.0	175	1	-	-	-	-

Patients were treated prior and/or after initiation of Bevacizumab treatment with focal laser or intravitreal triamcinolone. Hemodilution was made within the first weeks of diagnosis. No additional treatment was applied between the first injection and the subsequent visit 4 to 6 weeks later. Time points are represented in months (m) or days (d) prior to (negative values) or after (positive values) the first Bevacizumab injection.

### Short-term effects of Bevacizumab

**Figure 1:**
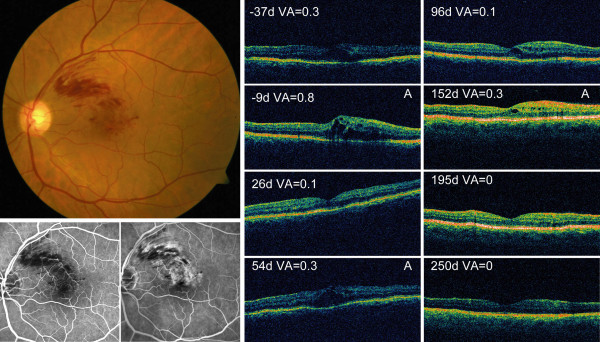
**Example of a patient treated with Bevacizumab for branch retinal vein occlusion (BRVO) associated macular edema**. Left, fundus photograph and fluorescein angiography show a non ischemic macular BRVO with macular edema in the left eye. Right, follow up of visual acuity and OCT for the same patient. As the edema increased and visual acuity worsened Bevacizumab treatment was indicated (day-9) and performed (day 0). Time course shows rapid improvement of visual acuity and macular edema, which was followed twice by a recurrence requiring further Bevacizumab injections (marked with Inj).

**Figure 2:**
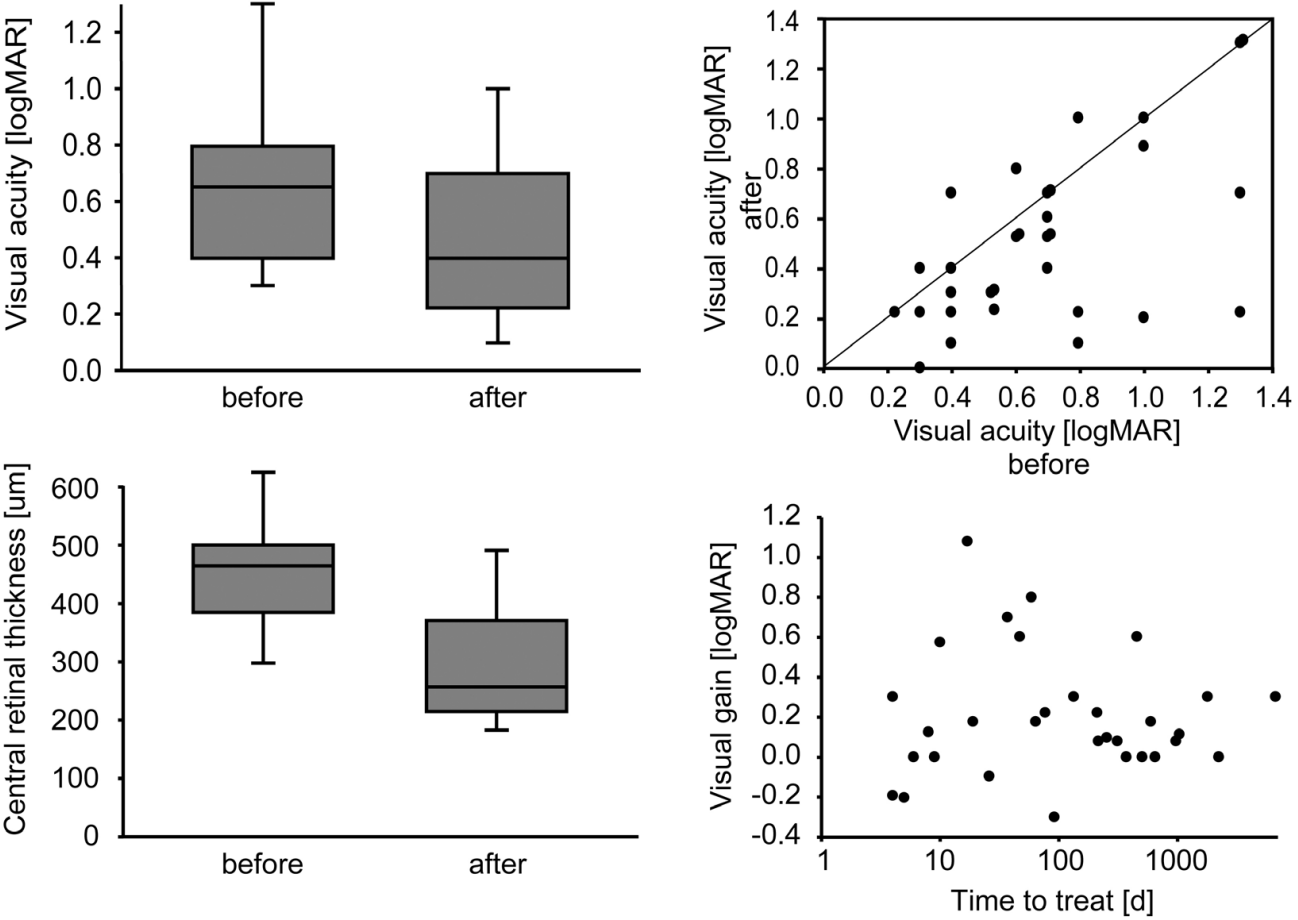
**Short term effects of Bevacizumab on visual acuity and retinal thickness**. Top left panel, box plot of logMAR visual acuity significantly improves 4 to 6 weeks after Bevacizumab injection. Boxes indicate 25 and 75 percentile, whiskers indicate 10 and 90 percentile, line within the box marks the median. Visual acuity and retinal thickness at the time the indication for treatment was made (before) is compared to measures taken within 6 weeks after injection (after). Top right panel, raw data for individual patients show that some patients respond with improved visual acuity whereas others fail to improve. Bottom left, box plot shows significant decrease of CRT after Bevacizumab application. Bottom right, correlation of visual gain and temporal delay from diagnosis of BRVO until first Bevacizumab treatment shows that improvement of visual acuity occurs independently of the age of BRVO. Note that time axis is logarithmic.

Within 6 weeks after injection BCVA and CRT were determined. The mean follow up interval was 30 ± 11 days. BCVA was 0.68 ± 0.3 and 0.5 ± 0.35 logMAR, before and after injection respectively (p < 0.01, paired t-test). CRT decreased from 454 ± 117 μm to 305 ± 129 μm (p < 0.01, paired t-test). 41% of all injected eyes showed a visual improvement of at least 2 lines after injection. BCVA remained unchanged in 53% and decreased by ≥ 2 lines in 6%. An example of a patient with favorable outcome is shown in figure 1. To investigate whether the temporal delay from diagnosis until treatment initiation altered the outcome, we correlated the time span from diagnosis of BRVO until the first injection of Bevacizumab with the visual gain, i.e. improvement of visual acuity 4 to 6 weeks after the first injection. There was no significant correlation found (Rho = 0.01, p = 0.9, Spearman's Rho Test, figure 2). Thus, in our group of patients the duration of presence of the BRVO seems not to influence visual gain induced by Bevacizumab treatment.

### Long-term effects of Bevacizumab

Repeated injections were performed if macular edema recurred after an initial decrease. 53% of all patients had only one injection at the time of analysis, 28% had two and 18% more than two. A mean of 1.7 injections were made per patient with an interval between injections of 92 days, range 42 – 306 days. Treatment was stalled when vision and CRT reached steady state, i.e. did not deteriorate more than one line (corresponding to maximally 0.3 logMAR units) and/or retina did not thicken more than 10% compared to the measures obtained six weeks after the last Bevacizumab injection.

**Figure 3:**
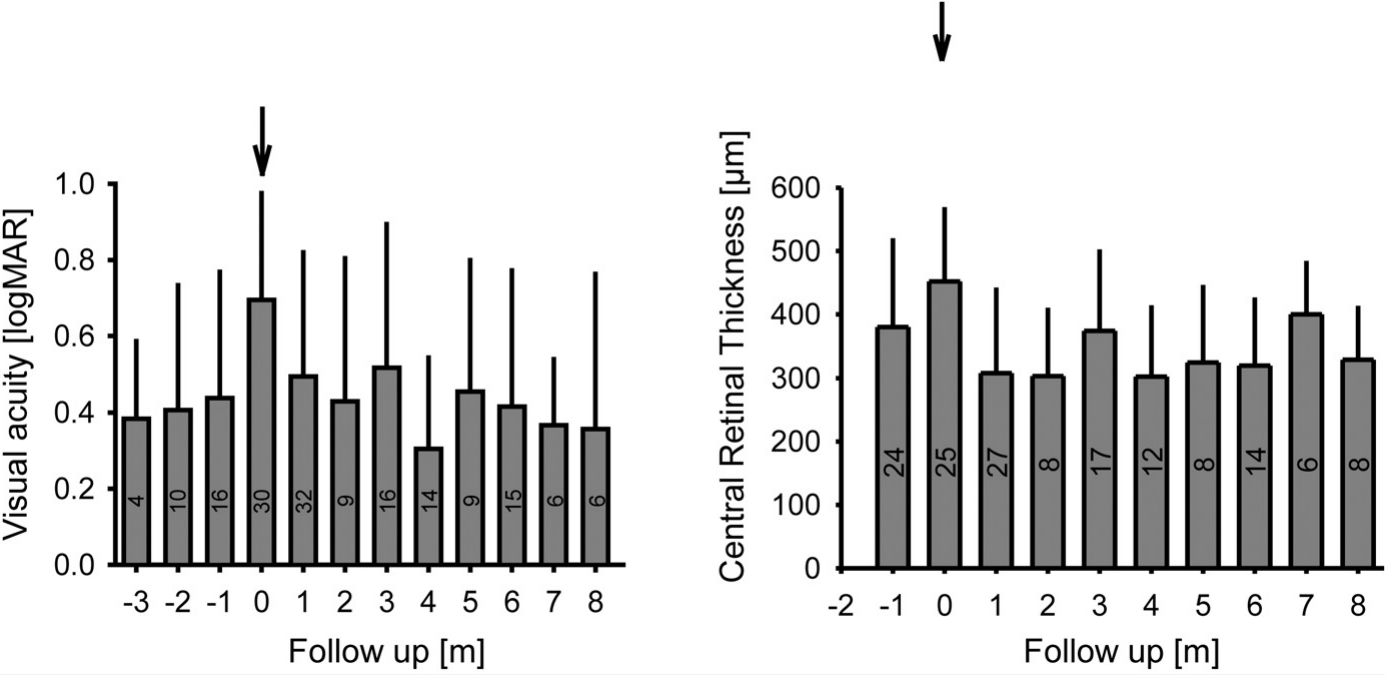
**Follow up of visual acuity and central retinal thickness**. Binned and temporally aligned data shows that Bevacizumab injection is preceded by a recent worsening of both visual acuity and CRT. Treatment results in a rapid and lasting improvement for both, requiring several injections though. Bin size is shown within bars; whiskers on bars represent standard deviation.

Follow-up data ranging from 27 – 418 days, median 170 days were evaluated. For each patient a timeline was calculated. Timelines were aligned for all patients with day 0 defined as time of first Bevacizumab injection. Data was binned with monthly intervals in order to obtain graphs as shown in figure 3. One month prior to first injection, visual acuity was better than at the time the decision for treatment with Bevacizumab was made. Bevacizumab led to a rapid improvement of visual acuity that was lasting for several months. This was paralleled by a similar time course for OCT measures, which showed increased CRT at day 0 and a lasting decrease thereafter.

## Discussion and conclusion

We show that intravitreal Bevacizumab treatment for macular edema induced by BRVO leads to a fast and significant improvement of visual acuity and a concurrent decrease of CRT. Similar results were previously accomplished with intravitreal administration of triamcinolone[3]. In contrast to triamcinolone however we did not observe any of the severe and rather frequent ocular side effects of intravitreal triamcinolone administration such as rise in intraocular pressure or cataract formation.

Our preliminary data on longer term effects suggest that the rapid beneficial effects of Bevacizumab last for several months, although individual patients require repeated injections. A natural course of the disease could not be predicted since the retrospective study design did not include a control group.

Our findings are in agreement with published case series showing that Bevacizumab treatment leads to improved visual function and decreased CRT in patients suffering from BRVO with macular edema [5-11]. These studies show a beneficial effect of Bevacizumab on both visual acuity and CRT. However, they reveal a great variability in injection frequencies, follow up time and treatment interval. Whereas Pai *et al.*[6], Stahl *et al.*[8] and Jaissle *et al.*[5] show only small case series, a study by Rabena *et al.*[7] with 27 patients is comparable to our study. Rabena *et al.*[7] had more dry patients after a single injection (80%), whereas in our hands 45% of the patients required more than one application of Bevacizumab. Three larger series published in 2008 come to similar conclusions: both prospective[9,10] and retrospective[11] analysis demonstrate that repeated applications of Bevacizumab are necessary for long lasting results on visual acuity and CRT.

Anti-VEGF treatment is not limited to either Bevacizumab or BRVO: Campochiaro *et al. *demonstrated that Ranibizumab, similar to Bevacizumab, leads to improved visual acuity and CRT in BRVO[12]. In addition to treating BRVO, Bevacizumab was used for central retinal vein occlusion: visual acuity, retinal thickness(see for example [13]) and ERG amplitudes[14] improved after Bevacizumab treatment in patients with macular edema due to central retinal vein occlusion.

Despite an increasing body of evidence for the efficacy of anti-VEGF treatment for macular edema associated with vein occlusion, an optimal treatment regime is yet to be determined. Indication for treatment and retreatment is based on deterioration of BCVA and retinal thickness, whereas an optimal treatment plan would act prophylactically. In our series, visual acuity was determined about 1 week prior to actual intravitreal injection. If we assume that visual deterioration continued during this interval, then our data represents rather an underestimate of the real efficacy of Bevacizumab.

Even though an optimal treatment regimen is as yet unclear, our data suggests that treatment should be initiated even if BRVO has been present for a while: in our patients the temporal delay from diagnosis to treatment initiation did not influence visual outcome (figure 2). This might be explained by the fact that the indication for treatment was based on the development of macular edema, which itself evolves with a temporal delay. Our dataset however did not allow a clarification of this issue. Similar observations were made in recently published series [9-11].

In conclusion our results suggest anti-VEGF treatment is a valuable therapy for BRVO-associated macular edema. Randomized clinical trials are now required for further evaluation.

## Competing interests

The authors declare that they have no competing interests.

## Authors' contributions

JF and UW conceived the study and participated in its design. DB and SW helped in interpretation of the data, were involved in scientific discussions and helped improve the manuscript. CT helped with data collection and writing, MA collected most of the data, drafted the manuscript and performed all analysis.

## Pre-publication history

The pre-publication history for this paper can be accessed here:


